# “Subsidized Journals,” “Canned Editorials”

**Published:** 1906-06

**Authors:** 


### EDITORIAL NOTES AND COMMENTS.

The Business office of THE JOURNAL-RECORD is No. 1007-1008 Century Building.

The Editorial office is 318-319-320 The Prudential.

Address all Business communications to Dr. M. B. Hutchins, Mgr.

Make remittances payable to THE ATLANTA JOURNAL-RECORD OF MEDICINE.

On matters pertaining to the Editorial and Original Communications address Dr. Bernard Wolff, Atlanta.

Reprints of original articles will be furnished at cost price. Requests for the same should always be made *on the manuscript*.

We will present, post-paid, on request, to each contributor of an original article, twenty (20) marked copies of THE JOURNAL-RECORD containing such article.

### "SUBSIDISED JOURNALS," "CANNED EDITORIALS."

WE publish below some remarks by *Journal of American Medical Association* of May 26, 1906. It omits our editorial, which preceded the matter complained of. We reproduce it, to supply the defect. They say we omitted a short paragraph of Antikamnia letter. Why did it omit our editorial, if honest in their criticism? Did not *The Journal of American Medical Association* omit one part of the Collier's exposure of the "Ozone" preparations? The fleeing thief in a city crowd often escapes by yelling "stop, thief," as if he were pursuer instead of pursued. The cry of "sudsidised" is a subterfuge and libelous.

As to "canned editorials," we need neither canned editorials, canned morality, nor canned ethics. Some of them might be embalmed. Nobody really thought "a letter" was from a disgruntled subscriber. None of our friends believe us "subsidised." The really excellent, though at times hasty, *Journal of the Ameri-*

*can Medical Association* has power for wonderful good if properly conducted.

### THIS TIME IT IS AN ANONYMOUS LETTER.

The ATLANTA JOURNAL-RECORD OF MEDICINE takes it up differently. The antikamnia letter in this instance is put into the editorial department, double leaded, but under the caption "A Letter." It is, of course, not signed; the readers are kept in blissful ignorance of the author; they might naturally conclude that some disgruntled subscriber had gone back on his fellow-practitioners and had turned to the defense of the "patent-medicine" men. One short paragraph in the body of the letter is omitted, and the concluding paragraph in which the journal is asked "to assist" is kept from the reader.

By the way, this number of the ATLANTA JOURNAL-RECORD OF MEDICINE devotes sixteen editorial pages to the defense of nostrums. Besides the antikamnia letter it quotes nearly all of the pamphlet sent to newspapers entitled "Legislative Schemes of the American Medical Association"; also another article circulated by the Proprietary Association of America, "The Mendacious Campaign Against Domestic and Proprietary Remedies," which is an attack on *Collier's* "Great American Fraud" articles.

### IF NEWSPAPERS, WHY NOT MEDICAL JOURNALS.

The "patent-medicine" men's association has a very effective press committee, which prepares and furnishes to such newspapers as are under the control of that association, material to be used to influence public opinion in favor of "patent medicines." As this material is usually attractively presented and readable, it is a great temptation to editors to print it as received rather than to transpose the arguments into their own words. This is the reason that the same editorials defending "patent-medicine' appear in newspapers all over the country. This press committee, therefore, is a great labor-saving device for the weak newspapers that are willing to be used by their supporters.

If newspapers can do this—can use as original the material furnished them—why can not medical journals? They can. They do. But as there is no real central press committee to act for the "ethical" nostrum manufacturer, some of them are doing it for themselves, as the Antikamnia Company, with its letter to the medical journals. As a member of the Proprietary Association of America, that company learned how to do it.

And the medical journals are even more willing to aid the "ethical" nostrum men than are the newspapers to aid the "patent-medicine" men. As the former have no press committee, certain medical journals are working up their own material, and then sending it around for others to copy. For instance, we have before us "advanced sheets of editorial pages of *Medical*

*Sentinel,* Portland, Ore., for May, 1906, Henry Waldo Coe, M.D., Editor." It contains six editorials for the faithful to copy: "The American Medical Association Gang," "A Medical Bureaucracy," "Directors Who Do Not Direct," "The Self-Effacement of Local Medical Journals," "Is the A. M. A. Ring 'Unbustable'," and "The House of Delegates of the A. M. A."

The faithful will copy.—*Journal Americal Medical Association,* May 26, 1906.

### AN UGLY FIGHT.*

We publish herewith liberal quotations from the people who have become stirred up by the fight of the *Journal of the American Medical Association,* and other publications against the patent and proprietary medicines.

Both sides are in part right, and both are in many respects wrong, and waging a nasty warfare which, as extracts show, is catching the doctor between and making him the real sufferer. There was never any good in going to extremes, and these controversies simply engender bitterness and arouse enmity, the effect of which will be hurtful to all concerned and defeat any honest efforts towards needed reforms.

It is not true that all proprietaries are bad. It is not proper for doctors to say too much about self-prescribing or drug-store practice of medicine, for it gives the drug men unfair means of defense. Some proprietaries are better than the average doctor can prescribe and have properly put up by the druggist. As to people going and buying a proprietary direct, so can they do with standard drugs, and, further, one prescription from a doctor is often filled and refilled and even passed on to the neighbors.

On the other hand, if the manufacturers and druggists are to do the prescribing for the sick, why not abolish medical colleges, repeal medical laws, and let every one be a doctor? Why should men devote years to the study of medicine and spend, often, other years of poverty in an effort to heal the sick if the drug people believe that every man should be his own doctor?

As said before, the doctor is the one who is going to suffer through this bitter controversy, and it behooves those who claim to be fighting for the doctor to bear this in mind.

Misrepresentations and false statements are already rife, each side is going to extremes, and unless a more conservative and consistent attitude is taken more harm than good is likely to result. No class should have, or desire, a monopoly, no one of decent honesty should wish to profit by the ills of humanity and none but a ghoul would so profit. Let the fight be fair, stop this unholy war and get together on a basis of "live and let live"—with honor.

---

*This Journal, May, 1906.

